# H3Africa AWI-Gen Collaborative Centre: a resource to study the interplay between genomic and environmental risk factors for cardiometabolic diseases in four sub-Saharan African countries

**DOI:** 10.1017/gheg.2016.17

**Published:** 2016-11-22

**Authors:** M. Ramsay, N. Crowther, E. Tambo, G. Agongo, V. Baloyi, S. Dikotope, X. Gómez-Olivé, N. Jaff, H. Sorgho, R. Wagner, C. Khayeka-Wandabwa, A. Choudhury, S. Hazelhurst, K. Kahn, Z. Lombard, F. Mukomana, C. Soo, H. Soodyall, A. Wade, S. Afolabi, I. Agorinya, L. Amenga-Etego, S. A. Ali, J. D. Bognini, R. P. Boua, C. Debpuur, S. Diallo, E. Fato, A. Kazienga, S. Z. Konkobo, P. M. Kouraogo, F. Mashinya, L. Micklesfield, S. Nakanabo-Diallo, B. Njamwea, E. Nonterah, S. Ouedraogo, V. Pillay, A. M. Somande, P. Tindana, R. Twine, M. Alberts, C. Kyobutungi, S. A. Norris, A. R. Oduro, H. Tinto, S. Tollman, O. Sankoh

**Affiliations:** 1 Sydney Brenner Institute for Molecular Bioscience, Faculty of Health Sciences, University of the Witwatersrand, Johannesburg, South Africa; 2 Division of Human Genetics, National Health Laboratory Service and School of Pathology, Faculty of Health Sciences, University of the Witwatersrand, Johannesburg, South Africa; 3 Department of Chemical Pathology, National Health Laboratory Service, Faculty of Health Sciences, University of the Witwatersrand, Johannesburg, South Africa; 4 Navrongo Health Research Centre, Navrongo, Ghana; 5 MRC/Wits Developmental Pathways for Health Research Unit, Faculty of Health Sciences, University of the Witwatersrand, Johannesburg, South Africa; 6 Department of Medical Science, Public Health and Health Promotion, School of Health Care Sciences, Faculty of Health Sciences, University of Limpopo, Polokwane, South Africa; 7 MRC/Wits Rural Public Health and Health Transitions Research Unit (Agincourt), School of Public Health, Faculty of Health Sciences, University of the Witwatersrand, Johannesburg, South Africa; 8 School of Public Health, Faculty of Health Sciences, University of the Witwatersrand, Johannesburg, South Africa; 9 Clinical Research Unit of Nanoro, Institut de Recherche en Sciences de la Sante, Ouagadougou, Burkina Faso; 10 African Population and Health Research Center, Nairobi, Kenya; 11 School of Electrical and Information Engineering, University of the Witwatersrand, Johannesburg, South Africa; 12 INDEPTH Network, Accra, Ghana; 13 Department of Mathematics and Statistics, Njala University, Njala, Sierra Leone

**Keywords:** AWI-Gen, body composition, cardiometabolic disease, diabetes, disease outcome, environmental risk factors, genomic studies, H3Africa, health transition, hypertension, NCD, non-communicable disease in Africa, obesity, stroke

## Abstract

Africa is experiencing a rapid increase in adult obesity and associated cardiometabolic diseases (CMDs). The H3Africa AWI-Gen Collaborative Centre was established to examine genomic and environmental factors that influence body composition, body fat distribution and CMD risk, with the aim to provide insights towards effective treatment and intervention strategies. It provides a research platform of over 10 500 participants, 40–60 years old, from Burkina Faso, Ghana, Kenya and South Africa. Following a process that involved community engagement, training of project staff and participant informed consent, participants were administered detailed questionnaires, anthropometric measurements were taken and biospecimens collected. This generated a wealth of demographic, health history, environmental, behavioural and biomarker data. The H3Africa SNP array will be used for genome-wide association studies. AWI-Gen is building capacity to perform large epidemiological, genomic and epigenomic studies across several African counties and strives to become a valuable resource for research collaborations in Africa.

## Background and introduction

Adult onset non-communicable diseases (NCDs) are responsible for 38 million deaths annually, of which 14 million occur between the ages of 30 and 70 years, with 85% of the latter occurring in low and middle income countries [1]. The World Health Organization's NCD Action Plan (2013–2020) has set the target of a 25% reduction in premature mortality from NCDs by 2025 [2]. It is therefore timely to focus on an NCD research agenda for sub-Saharan Africa (SSA). One of the main drivers of the increase in cardiometabolic diseases (CMDs) on the continent is obesity [3] and therefore a better understanding of the role of genomic, environmental and behavioural factors in modulating body fat distribution is necessary. Furthermore, there are some interesting differences in disease epidemiology and pathophysiology for NCDs between SSA and high-income countries. For example, in most African countries women have a much higher prevalence of obesity than men, whereas the prevalence of obesity in the developed world is more equally distributed across the sexes [4]. In addition, the waist circumference cut point used to diagnose the metabolic syndrome in SSA appears to differ from that used in other populations [5].

A health and demographic transition is at different stages in countries across Africa and varies between rural and urban communities [4]. The country-specific population data, NCD mortality and the prevalence of risk factors in adults for Burkina Faso, Ghana, Kenya and South Africa are shown in Table 1. South Africa appears the farthest along the transition, with the highest proportion of the population (62%) living in urban areas and the highest rates of obesity with 31.3% of individuals with a body mass index (BMI) ≥ 25 (World Health Organization: Non-communicable Diseases Country Profiles, 2014). There is considerable within-country variation, which can be stratified along an urban:rural divide, where poverty is concentrated in rural settings, or according to socioeconomic gradients, or ethnolinguistic groups.

**Table 1.** Population data, non-communicable disease (NCD) mortality and adult risk factors in Burkina Faso, Ghana, Kenya and South Africa^a^

Country	Burkina Faso			Ghana			Kenya			South Africa		
Total population	16 460 000			25 366 000			43 178 000			52 386 000		
Income group	Low			Lower middle			Low			Upper middle		
Proportion of population 30 to 70 years	25.3%			30.9%			27.3%			38.3%		
Total deaths estimated to be attributable to NCDs	23%			42%			27%			43%		
NCDs mortality												
Cardiovascular disease	12%			18%			8%			18%		
Diabetes	2%			2%			1%			6%		
Cancer	4%			5%			7%			7%		
Chronic respiratory disease	2%			2%			1%			3%		
Others	12%			14%			9%			10%		
Premature mortality due to NCD^b^	24%			20%			18%			27%		
Adult NCD risk factors	Tot	W	M	Tot	W	M	Tot	W	M	Tot	W	M
Current tobacco smoking % (2011)^c^	–	–	–	10	7	14	13	<1	26	18	8	28
Total alcohol per capita consumption (2010)^d^	6.8	2.8	11.2	4.8	1.9	7.8	4.3	1.3	7.4	11.0	4.2	18.4
Raised blood pressure % (2008)^e^	29.4	28.8	29.9	27.3	26.5	28.2	28.7	26.7	30.7	33.7	32.4	35.2
Obesity % (2008)^f^	2.3	3.0	1.5	7.5	10.9	4.1	4.2	6.2	2.1	31.3	41.0	21.0

–, Data not available; W, women; M, men; Tot, mean for men and women.

a World Health Organization: Non-communicable Diseases (NCD) Country Profiles, 2014.

b The probability of dying between ages 30 and 70 years from the four main NCDs.

c Current tobacco smoking (2011): the percentage of the population aged 15 or older who smoke any tobacco products.

d Total alcohol per capita consumption, in litres of pure alcohol (2010): consumption of pure alcohol (recorded and unrecorded) per person aged 15+ during one calendar year.

e Raised blood pressure (2008): the percentage of the population aged 25 or older having systolic blood pressure ≥ 140 mmHg and/or diastolic blood pressure ≥90 mmHg.

f Obesity (2008): the percentage of the population aged 20 or older having a body mass index ≥30 kg/m^2^.

To gain a more comprehensive understanding of susceptibility to CMDs in SSA, it is necessary to study the genetic variation and gene–environment interactions that could affect risk, and to develop a region-specific knowledgebase to support the development of appropriate and sustainable prevention strategies. It is thus both timely and relevant to develop large African population cohorts for which genomic, demographic, environmental, behavioural and anthropometric data, as well as blood and urine biomarkers are available. Such an initiative is the subject described here.

The Africa Wits-INDEPTH partnership for Genomic Studies (AWI-Gen) is an NIH funded Collaborative Centre of the Human Heredity and Health in Africa (H3Africa) Consortium [6]. It is a strategic partnership between the University of the Witwatersrand, Johannesburg (Wits), and the International Network for the Demographic Evaluation of Populations and Their Health (INDEPTH), and leverages their respective research strengths. It capitalises on the unique characteristics of existing Health and Demographic Surveillance System (HDSS) centres and the Developmental Pathways for Health Research Unit (DPHRU), that have longitudinal cohorts in urban (Soweto and Nairobi) and rural (Navrongo, Nanoro, Agincourt and Dikgale) settings. They offer established research infrastructure, including long-standing community engagement (CE), trained fieldworkers, and detailed longitudinal demographic data, and in some cases, phenotypic data focusing on obesity and cardiometabolic health. A key strength is the representation of the geographic and social variability among African populations. In addition, Wits University contributes expertise in population genetics, genome-wide disease association studies and bioinformatics.

## AWI-Gen aim and objectives

AWI-Gen aims to study the long-term health consequences of rapidly changing environmental and demographic conditions in the context of African genome diversity, and to inform public health interventions to mitigate the rising burden of NCDs [6].

The AWI-Gen vision is to establish a set of longitudinal research cohorts for CMDs in populations from countries across Africa with different socioeconomic, ethnic, climatic and historic backgrounds in conjunction with harmonised phenotype, environmental exposure, biomarker and genomic data to examine both vulnerability to disease and disease outcomes. AWI-Gen will contribute to building infrastructure across participating centres, which includes molecular biology laboratories and biorepositories, and to develop and enhance skills for planning, executing and analysing data on the African continent.

The research project has three broad objectives, as follows:

1. To build capability for genomic research in the centres by providing opportunities to enhance and develop skills. The centres have expertise in data collection and management, epidemiological research, and biostatistical analyses, but few have had an opportunity to do genetic and genomic studies. We provide a cross-disciplinary research environment including genomics and bioinformatics, and promote study opportunities for postgraduates, and skills development for both emerging and senior researchers.
2. To understand the population structure and genetic architecture among the study participants to inform analysis strategies and to evaluate impact across the ethnolinguistic groups. In our study, the urban communities are particularly complex as they represent a convergence of the ethnolinguistic groups of a country and neighbouring regions, due to migration in pursuit of employment opportunities.
3. To investigate independent and interacting genomic, environmental and behavioural contributions to body composition and body fat distribution (height, weight, hip and waist circumference, subcutaneous and visceral fat) which are major risk factors for CMDs.

## Participating centres

**Fig. 1.**
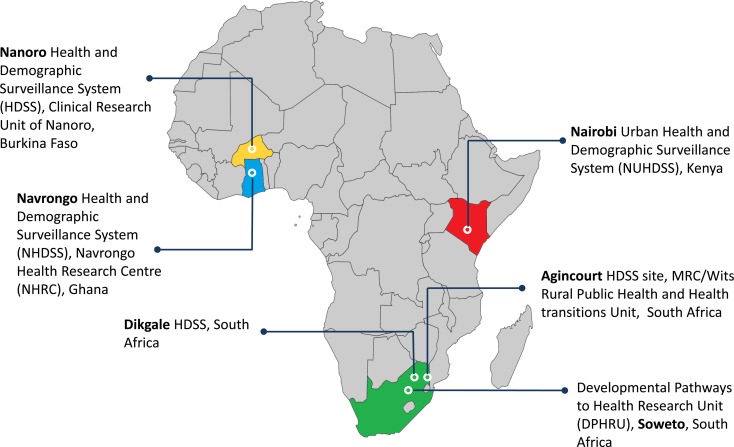
Map showing the locations of the catchment areas for the AWI-Gen study in Africa.

The AWI-Gen study participants are drawn from five INDEPTH member HDSS centres across the African continent, ensuring a balance of west, east and southern African populations from rural and urban settings. These centres are located in Nanoro (Burkina Faso) [7], Navrongo (Ghana) [8], Nairobi (Kenya) [9], Agincourt (South Africa) [10] and Dikgale (South Africa) [11]. The sixth centre is in Soweto and is coordinated within the DPHRU, located at the Chris Hani Baragwanath Hospital, South Africa [12]. The geographic regions of these centres are shown in Fig. 1, and a brief historical summary and research focus of each are provided in Table 2.

**Table 2.** AWI-Gen Study Centres

Centre, country	Brief history	Research focus
Nanoro, Burkina Faso	The Nanoro HDSS was established at the Clinical Research Unit of Nanoro (CRUN) in 2009 and covers a population of 63 000 inhabitants in 24 villages and 8 peripheral health facilities. Vital events are collected through household visits carried out every four months	The research focus of the CRUN was traditionally malaria, infectious diseases and community-based interventions. Recently, it's research portfolio has been expanded to include NCDs that are on the rise in SSA. Up until now, the AWI-Gen project is the largest study on NCDs to be conducted by the CRUN
Navrongo, Ghana	The Navrongo Health Research Centre (NHRC) started in 1988 as a field site for the Ghana Vitamin A Supplementation Trial and was upgraded into a Health Research Centre in 1992 by the Ministry of Health of Ghana. Currently the major research areas are in biomedical and the social sciences. In addition, the Centre has a functional Health and Demographic Surveillance System (HDSS) which was established in 1993; the Navrongo HDSS does routine monitoring on health and demographic dynamics including pregnancies, births, morbidities, deaths, migrations, marriages and vaccination coverage. Other important support units include clinical trials, research laboratories, data centre and general administration. The Centre has an Ethics Review Board that has Federal Wide Assurance	The biomedical research focuses on clinical trials, maternal and child health, environmental health, mental health, pathogen and vector studies and genomic research. The social science research themes include adolescent and adult health, health systems, community health, health promotion and education, and ethics and behavioural studies
Nairobi, Kenya	The Nairobi Urban Health & Demographic Surveillance System (NUHDSS) was the pioneer urban-based Health and Demographic Surveillance System in SSA established in 2002. The platform was set up by the African Population and Health Research Centre in two informal settlements of Korogocho and Viwandani in Nairobi. Currently the NUHDSS follows a population of about 75 000 individuals in approximately 24 000 households in the two slum communities. The main objective of the site is to provide a longitudinal platform for investigating linkages between urban poverty and wellbeing outcomes including health, demography, and schooling. In addition to the routine data, it has progressively continued to provide a robust platform for nesting several studies examining the challenges of rapid urbanisation in SSA and associated health and poverty dynamics	A robust research program on cardiovascular disease risk factors is nested in the NUHDSS. Participation in AWI-Gen provides an opportunity to build on this work and grow capacity in biomedical approaches to understanding the role of environmental and social factors in health
Agincourt, South Africa	The Agincourt HDSS is a South African Medical Research Council and Wits University research unit. Located in rural northeast South Africa close to the Mozambique border, it was established in 1992 to support decentralised district and sub-district health systems development as South Africa transitioned from the apartheid era to democracy. Work since then has documented and responded to rapid and complex health, population and social transitions	Currently there are major trials and observational studies across key stages of the life course, including HIV and NCD prevention in adolescents and their offspring, interactions of CMDs and HIV in middle-aged and older adults, integrated management of hypertension and other chronic conditions in primary health care facilities, community mobilisation interventions to address gender norms and enhance linkage to care, key aspects of ageing including cognitive function, and social determinants including migration and health
Dikgale, South Africa	The Dikgale HDSS was started in 1996, is situated about 40 km north-east of Polokwane, the capital of the Limpopo Province, and includes approximately 7000 households covering a population of about 36 000. An annual survey of life events, including verbal autopsies to establish cause of death is conducted	The research focus is on chronic diseases in a rural setting with an emphasis on nutrition, physical activity and biochemical markers of disease risk
Soweto, South Africa	MRC Developmental Pathways for Health Research Unit (DPHRU) started in 2010 and is based at Chris Hani Baragwanath Academic Hospital in Soweto. DPHRU builds upon long-term ties with the Soweto community. A flag-ship project of DPHRU is the Birth to Twenty cohort, a birth cohort established in 1990 that has followed over 3000 mothers and babies born in the Soweto-Johannesburg area for 25 years. They are now following three generations from this cohort	The research activities of DPHRU align with two national priorities: improving maternal and child health, and tackling obesity and metabolic disease risk

## Rationale and methodology: AWI-Gen trait-association study

AWI-Gen is a population-based cross-sectional study that includes over 10 500 unrelated participants of 40–60 years of age, including both men and women, whom are resident in the areas served by the HDSS centres. Exclusion criteria are: closely related individuals, pregnant women, and recent immigrants (<10 years) into the communities. There was no selection based on body composition, infection or disease history.

### Questionnaire

An extensive paper-based questionnaire was administered to each participant by a trained field worker or clinician, except in Agincourt where the interview was done using a computer-assisted personal interviewing system. The questionnaire contained three main sections: Demography, Health History and Anthropometry. The board categories of data collected in the first phase of the AWI-Gen study are listed in Table 3.

**Table 3.** Categories of AWI-Gen data collected

Category	Variable
Socio-demographic	Age
	Sex
	Country
	Home language
	Self-reported ethnicity/tribe
	Family composition
	Pregnancy status
	Marital status
	Employment
	Level of education
	Household attributes for social economic status (SES)
Health History (Cardiometabolic risk factors and general health)	Diabetes, stroke, hypertension, angina, heart attack, congestive heart failure, high cholesterol, thyroid disease, kidney diseases, breast/cervical/prostate/other cancers, asthma or reactive air diseases, weight problem/obesity
Anthropometry	Weight
	Height
	Blood pressure
	Pulse
	Waist circumference
	Hip circumference
Ultrasonography	Visceral fat
	Subcutaneous fat
	Carotid intima media thickness (cIMT)
Environmental	Tobacco use
	Alcohol use
	Drug use
	Diet
	Exercise/general physical activity questionnaire (GPAQ)
	Exposure to pesticides
Infection history	Malaria, Tuberculosis, HIV

### Sample collection, storage and availability

Fasting venous blood and spot urine samples were collected and processed for biomarker assays according to Standard Operating Procedures (SOPs) developed for AWI-Gen. Processed samples were frozen and shipped on dry ice to the Sydney Brenner Institute for Molecular Bioscience (SBIMB) Biobank at Wits in Johannesburg, where DNA was extracted and a preliminary set of biomarkers measured, as listed in Table 4. DNA aliquots were sent to the H3Africa Biorepository at the Clinical Laboratory Services (CLS) in Johannesburg and an aliquot was returned to the respective study centre. Aliquots of serum, plasma and urine were frozen at −80 °C and stored for future analyses that will enrich the dataset, and enable additional research. Banked biospecimens will be available through the H3Africa Data and Biospecimen Access Committee (DBAC) from dedicated H3Africa Biorepositories (http://h3africa.org/), or through direct collaboration with AWI-Gen.

**Table 4.** Blood and urine biomarkers tested in AWI-Gen participants

Sample type	Variable
Fasting blood (serum)	HDL
	LDL
	Total cholesterol
	Total triglycerides
	Fasting insulin
Fasting blood (plasma)	Fasting glucose
Urine	Albumin
	Total protein
	Creatinine

### Infectious diseases as co-morbidities

Infection history and treatment for human immunodeficiency virus (HIV), malaria and tuberculosis were documented in the regions where these infections are endemic. Thus, in the four study centres in east and southern African, HIV testing was offered to participants on a voluntary basis. Information on HIV status is important as both the infection with the virus, and its therapy, are major modifiers of body fat distribution and may influence blood biomarker levels [13, 14]. Malaria is endemic in West Africa and since almost all participants from this region will have been exposed during their lifetime, data were collected only to the extent of active malaria infection in the 2 months prior to enrolment.

### CMD risk factors

Obesity indicators, including BMI and waist-to-hip ratios, were calculated from anthropometric measurements; and visceral and subcutaneous fat were measured by ultrasound. Other indicators of CMD outcomes were measured, including blood pressure, carotid intima media thickness (cIMT), fasting blood glucose and insulin levels and fasting lipid profiles. These data provide measures for the prevalence of overweight (BMI ≥ 25), obesity (BMI ≥ 30), hypertension (systolic blood pressure >140 mmHg and/or diastolic blood pressure >90 mmHg and/or currently on treatment for hypertension), type 2 diabetes (fasting blood glucose >7 mm/l and/or receiving treatment for diabetes) and metabolic syndrome. In addition, the health questionnaire provides data on family history and environmental exposures (tobacco, alcohol, insecticides and other substances) as well as information concerning physical activity, limited dietary information, and socioeconomic status. Notably, all HDSS centres offered comprehensive historical data on mortality and cause of death ascertainment through the use of verbal autopsies [15].

The individual study centres were encouraged to enrich their AWI-Gen data with additional variables of interest, for example additional body composition and anthropometry measures (DXA scanning, skin fold thickness and more extensive nutrition data) and data on cognitive function. In addition, some centres collected information on food security, migration history and sociodemographic events.

### Genomic study

A genome-wide SNP genotype dataset will be generated for all participants using the H3Africa SNP array. It is enriched for common variation in multiple African populations. Data generated by these studies will be used both for genome-wide exploratory research to identify novel genetic associations, as well as hypothesis-driven research, including replication studies. The genetic association studies will focus initially on the body composition and anthropometric variables, particularly the levels of visceral and abdominal subcutaneous fat, and the blood and urine biomarkers as risk factors or determinants of cardiometabolic outcomes.

### Data analysis strategy and statistical power

In the first instance, an exploratory genome-wide association study (GWAS) will be performed using the H3Africa SNP array. This will provide a base from which to perform GWASs for multiple phenotypes related to body composition and cardiometabolic risk factors. Logistic regression will be used for categorical variables and linear regression for quantitative traits. This cross-sectional population study of approximately over 10 500 individuals (unselected for any disease phenotypes, but including individuals with common diseases of lifestyle like hypertension, stroke and diabetes) will be powered to detect significant associations. A model that assesses a continuous variable (e.g. BMI, blood pressure and lipid levels) in the independent individuals with a dominant genetic inheritance and an allele frequency of 0.04 will be >0.94 powered (*α* = 0.05) to detect a *β*_G_ (genetic effect) of 1.2. Likewise, an allele frequency of 0.20 will have >0.99 power to detect even a very small genetic effect. When analyses are done per site with only 2000 participants, the power is reduced to 0.67 to detect a *β*_G_ = 2 given an allele frequency of 0.04 and the power is >0.80 to detect a *β*_G_ > 1.32 given an allele frequency of 0.20. Calculations were performed using Quanto [16].

**Fig. 2.**
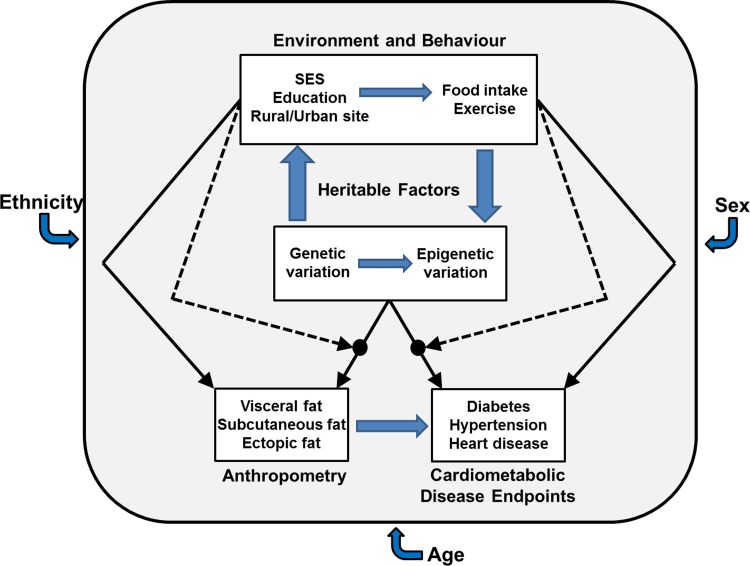
Complex interactions between the environment and behaviour, heritable factors and outcomes like anthropometry and biomarkers and their contribution to cardiometabolic endpoints are illustrated. These factors and interactions are further influenced by fixed non-modifiable factors including sex, age and ethnicity.

Complex modelling, including hierarchical regression analysis, will be used to examine the relationships between anthropometry, behaviour, biomarkers and genetic variants (Fig. 2) and to detect phenotype–environment, gene–gene, genotype–phenotype, genotype–environment interactions. These data will also be used to develop and validate genetic variation for potential Mendelian Randomisation approaches (reviewed in [14–16]) for studying risk factors in selected African populations.

## Rationale and methodology: AWI-Gen population structure study

### Rationale

Genomic diversity in various regions in Africa remains largely uncharacterised, despite some recent large-scale population genomic studies including African populations, most notably The HapMap Project, The 1000 Genomes Project, the African Genome Variation Project [17], and several smaller studies [18–21]. The goal of the AWI-Gen population structure study is to provide in-depth characterisation of genomic diversity in the regions where our study is being performed, and thereby contribute to a large, unbiased and systematic profile of sub-Saharan African population genomic diversity that will serve as a resource for genetic epidemiological studies.

African populations are genetically diverse and harbour signatures of inter- and intracontinental migration, genetic admixture, responses to the environment through natural selection and random drift [22]. The population structure study is expected to provide a better understanding of the way in which these factors influence susceptibility to disease and could contribute to predicting future health on the continent in the context of changing environments.

### Approach

Whole-genome sequencing (WGS) will be done on a subset of participants from under-studied ethnolinguistic groups in order to discover novel variants and to get a better understanding of common genetic variation. To date 60 individuals from the Mossi and Kassena ethnic groups in Burkina Faso and Ghana have been sequenced.

SNP genotyping arrays will be performed on all individuals and imputation will be done using reference whole genomes from closely related populations. These studies will shed light on genome architecture, population structure and genetic admixture in the different populations.

## Implementation strategies

### Ethics, CE and broad consent

The AWI-Gen study protocol, information sheet and informed consent documents, tailored to the local context and including translation into various local languages, was approved by the Human Research Ethics Committee of the University of the Witwatersrand (Protocol Number: M121029). In addition, each of the HDSS centres obtained ethics approval according to their respective institution and country-specific rules and regulations [23, 24]. The ethics approval process took an average of 4 months for most of the centres.

The HDSS centres each have an established CE process for introducing new research projects into their surveillance areas. A variety of approaches towards effective planning for CE have been highlighted in the H3Africa Guidelines for Community Engagement (http://h3africa.org/). The methods used in the AWI-Gen project, ranged from consultations with community leaders, community meetings and group discussions, as well as meetings with compound and household heads. Community engagement processes with recognised community structures provided an important opportunity for a multi-layered approach to share and reinforce information about the project. This approach allowed the research teams to work proactively with local partners to gain trust and to legitimise the project objectives. An evaluation of the CE strategies is being planned to inform future genomic studies.

One major challenge in obtaining consent for the AWI-Gen project was the difficulty in explaining genomics to potential participants and finding local terminologies and analogies to explain the science involved. The project followed the H3Africa Informed Consent Guidelines, which includes guidance on broad consent (http://h3africa.org/). Data and specimens were anonymised using study codes during sample processing, thereby respecting confidentiality. Only the individual research study centres maintain primary records that link participants to their personal identifiers. Participants have been assured that they may withdraw at any time with the understanding that their data can only be withdrawn for prospective studies.

### Approaches to capacity enhancement for genomic research

AWI-Gen and H3Africa aim to develop capacity for genomic research at multiple sites in Africa [25, 26]. The success of AWI-Gen hinges on effective training and skills development that ranges from CE to obtaining informed consent; field work and sample collection to processing in the laboratory; and from the laboratory through to successful genetic data generation and statistical, epidemiological and bioinformatics analyses and subsequent interpretation. To promote a deeper understanding of an ethical framework for genomic research in African countries we held a workshop for ethics review committee members in 2012 [17]. We also continue to hold workshops on AWI-Gen data management, data analysis and scientific writing with expert facilitators. The H3Africa pan-African Bioinformatics Network, H3ABioNet, has an extensive training programme for bioinformatics, which includes introductory modules, GWAS analysis and next generation sequencing data analysis (http://h3abionet.org/). Wits University is a node of H3ABioNet and is accredited for training in GWAS analysis. Postgraduate students are supervised and trained across our centres and we host research personnel and postdoctoral fellows for further training in the fields of biostatistics, epidemiology, genetic epidemiology, genomics and bioinformatics.

### AWI-Gen data management and sharing

Extensive quality control is the cornerstone of good data and knowledge generation. To ensure data quality we implemented a set of SOPs for the curation of questionnaire data, anthropometry, biomarkers and genomic data. This enhances the quality of the analyses and subsequent interpretation and dissemination of the findings. Software applications have been developed to store and manage the data.

Study data are being collected and managed using REDCap (Research Electronic Data Capture) [27] electronic data capture tools hosted at Wits. REDCap is a secure (HIPAA compliant), web-based application designed to support data capture for research studies, providing an intuitive interface for validated data entry, a set of audit trails for tracking data manipulation and export procedures, automated export procedures for seamless data downloads to common statistical packages and procedures for importing data from external sources.

Four centres have independent installations of REDCap running on dedicated Apple Mac Mini machines. This infrastructural setup was developed to address issues of poor internet connectivity in the majority of African countries, specifically in field sites where data were being collected. One centre uploaded data directly into REDCap on the Wits server and one used an electronic device to capture data that was later uploaded to REDCap.

AWI-Gen biospecimen data are stored and managed by the Laboratory Information Management System (LIMS) component of The Ark Informatics [28] at the SBIMB. The Ark Informatics is a suite of secure, integrated web-based tools that incorporate the majority of the functionality required to conduct a complex study or clinical trial.

The data will be managed and shared according to the policies and guidelines of the H3Africa Consortium [26] and in line with the informed consent of the participants and the ethics approvals for the study. There will be a process of managed access to phenotype and genetic data and biospecimens through approval from the H3Africa DBAC or through direct collaboration.

## Results

### Study characteristics and timeline

**Fig. 3.**
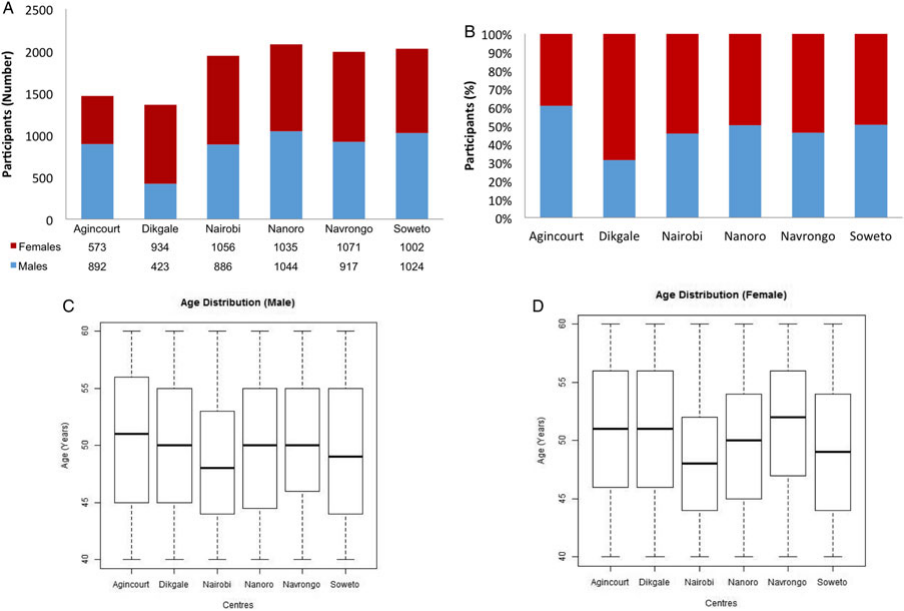
Characterisation of the AWI-Gen participants between the ages of 40 and 60 years showing sex distribution of participants as absolute numbers (A) and as a percentage (B) as recruited by each study center. Age distribution is shown for men (C) and women (D). Please note that participants outside the 40–60-year age range have not been included in the figures. The harmonisation with the HAALSI study at the Agincourt centre has resulted in the recruitment of additional participants over the age of 60 years.

Enrolment numbers and descriptive characteristics for 10 857 AWI-Gen participants between the ages of 40 and 60 years are shown in Fig. 3. The objective was to have roughly equal numbers of men and women (Fig. 3*a, b*). At the Dikgale centre more women were recruited due to the logistical challenges of recruiting men in a community where many men are working a distance from home and were reluctant to give up a weekend day to participate in the study. The age distribution is stratified for men and women for each centre (Fig. 3*c, d*). Our recruitment for AWI-Gen was completed in August 2016 and we have small numbers of individuals below the age of 40 and above the age of 60. These may be included in further research projects, as appropriate. The Agincourt study site purposely recruited individuals over the age of 60 years as part of a harmonization process with the *Health & Aging in Africa: Longitudinal Studies of INDEPTH Communities* (HAALSI) study.

Ethnicity is an important consideration in genetic studies and rather than delineating participants only according to country of origin, we requested information on self-reported ethnicity. A breakdown of the ethnic groups represented is shown in Table 5. In Soweto, the question of ethnicity was considered a potentially stigmatising question, and therefore participants were asked instead about their home language; language was therefore used as a proxy for ethnicity. Notably there is more ethnic homogeneity in the rural areas and more diversity and ethnic admixture in the urban settings.

**Table 5.** Self-reported ethnic distribution of AWI-Gen participants across the six study centres

South Africa					Burkina Faso		Ghana		Kenya	
Ethnicity	Agincourt	Dikgale	Soweto^a^	Total	Ethnicity	Nanoro	Ethnicty	Navrongo	Ethnicity	Nairobi
Tsonga	1244	57	138	1439	Mossi	1937	Kassena	1009	Kikuyu	698
BaPedi	31	1204	121	1356	Gourounsi	109	Nankana	876	Kamba	384
Zulu	33	12	674	719	Peulh	14	Bulsa	43	Luo	359
Sotho	69	11	327	407	Dagara	2	Mampruga	23	Luhya	316
Tswana	1	16	258	275	Dioula	3	Frafra	21	Kisii	62
Xhosa	2	6	191	199	Samo	3	Kantosi	7	Somali	51
Swati	56	2	63	121	Gourmatche	2	Mossi	3	Meru	30
Venda	5	26	63	94	Other^b^	5	Other^b^	4	Embu	21
Ndebele	2	17	21	40	Unknown^c^	4	Unknown^c^	2	Borana	8
Other^b^	1	3	12	16					Gari	3
Unknown^c^	21	3	158	182					Kalenjin	3
									Maasai	2
									Other^b^	5
Total	1465	1357	2026	4848	Total	2079		1988		1942

a In Soweto, language was used as a proxy for self-reported ethnicity.

b The category ‘other’ was used when there were only one or two individuals in a specific ethic category.

c The category ‘unknown’ was used when the person did not provide information on ethnicity.

Recruitment for the first phase of the AWI-Gen study ended in August 2016 and it is anticipated that the biomarker assays will be completed by the end of November 2016. The genome-wide genotyping data using the H3Africa SNP array will be generated in 2017.

### Strengths and limitations

A major strength of the study is that it is embedded in HDSSs of the INDEPTH Network, where each centre has built up deep relationships with the communities over many years, and in some cases decades. It is therefore possible to follow participants longitudinally and to have access to census data from the communities, as well as large additional datasets collected for others studies. There is ample opportunity for collaborative and nested research studies building on existing synergies and capitalising on the extensive networks of investigators with skills across multiple disciplines.

This study will provide an opportunity to collect base-line data on cardiometabolic risk factors, including relevant biomarkers and behaviours across communities in different African populations. Perceived limitations such as self-identified ethnicity and potential confounding in GWAS can be overcome by using principal component analysis and admixture programs to assess biological origins and affinities. Although this is a large study by African standards, the sample size has limitations in terms of the discovery of novel genetic associations with modest to small effects. It is, however, ideally suited for replication studies and for meta-analyses with other African cohorts that have collected similar data (for example, the H3Africa Cardiovascular Disease Working group [29]).

## Discussion

### Potential impact of AWI-Gen

One of the key aims of AWI-Gen is to determine the genetic and environmental contributions to body composition and body fat distribution, particularly visceral adiposity, in several African populations. Many studies have shown that this fat depot is the major anthropometric modulator of CMD risk [30] and that visceral fat mass has a strong level of heritability [31]. However, only two GWASs have investigated the genetic aetiology of visceral fat mass, using the gold standard methodology of computerised tomography (CT) scanning [32, 33]. The AWI-Gen study will represent the largest GWAS of visceral and subcutaneous adiposity, and is to date, the only study to perform such an analysis in several African populations. Due to the novel African genomic architecture, which exhibits low linkage disequilibrium and a high level of genetic diversity [17] it is likely that this GWAS will reveal new sequence variants associated with body fat distribution that would be located close to the actual causal variants.

The breadth of phenotypic data will allow AWI-Gen to perform GWAS on many important cardiometabolic traits including lipid levels, insulin resistance, cIMT as a proxy measure of atherosclerosis, blood pressure, glucose levels, metabolic syndrome and kidney function. The results of these analyses will provide both valuable baseline data as well as new data on the genetic determinants of CMDs in sub-Saharan African populations, and possibly identify some of the ‘missing heritability’ of complex, polygenic NCDs such as obesity, hypertension, dyslipidaemia and diabetes.

The results of the GWAS generated by AWI-Gen will be the proverbial ‘ears of the hippo’. The detailed phenotypes measured at all study sites will provide important information on the relationship between body fat distribution and cardiometabolic dysfunction in diverse African populations. Previous studies have shown that black African women are more insulin-resistant than BMI-matched white women but have less visceral fat [34, 35]. It is therefore important to analyse the relationship between visceral and subcutaneous fat mass and CMD risk factors in African countries with varying levels of obesity.

Data collected on alcohol and food intake, smoking, physical activity, education levels, and socioeconomic status, are all factors that have been widely studied and shown to be related to obesity and CMDs in other populations, but are less well studied in Africa. These data will allow us to study the influence of demographic, behavioural and environmental factors on obesity and NCD risk. Furthermore, HIV-status captured in South Africa and Kenya, which are the countries with the highest HIV prevalence within the AWI-Gen study sites, will allow an investigation of the relationship between HIV and treatment with NCDs and body fat distribution. A recent meta-analysis of data from SSA has shown that, as in populations of European ancestry, HIV infection and anti-retroviral therapy are associated with an increased CMD risk [36]. Whilst this study did suffer from limitations associated with combining different studies, each with its own methodology, AWI-Gen uses harmonised data from different study sites, each using the same standardised techniques, and blood biomarker data generated by the same laboratory.

The breadth of the phenotypic data collected for AWI-Gen, in combination with high-density genomic data, will enable us to analyse the interaction between demographic, socioeconomic, behavioural, genetic, anthropometric and cardiometabolic factors which will make it possible to isolate key correlates of body fat mass, body fat distribution and NCD risk in African populations (Fig. 2). Such an holistic approach will be essential for the development of effective public health intervention programmes for obesity and NCDs on the African continent.

AWI-Gen has started developing a collaborative network of researchers and clinicians from varied disciplines to grow stronger infrastructure in SSA to support NCD research. This research network is well positioned to engage in a wide spectrum of biomedical research and importantly would form the basis of several longitudinal cohorts across the continent.

The AWI-Gen team, data and bioresource will provide an integrated research platform for cross-disciplinary research for further genetic, genomic, epigenomic and environmental studies and will support future research and collaborations. The objective is to use this platform to contribute to strengthening healthcare systems and to improve health.

### Expansion, harmonisation, collaboration and future studies

AWI-Gen is developing an extensive African-specific dataset of highly phenotyped study participants with extensive biomarker and genome-wide genotype data. The phenotype and genetic data will be deposited in the European Genome-phenome Archive (EGA), and DNA samples, plasma, serum and urine are being stored at the SBIMB for future research. Data and DNA samples will be available through the DBAC of the H3Africa Consortium in accordance with its policies and guidelines. We wish to encourage collaborative research that will not only lead to potential benefit to the communities involved in the study, but will also provide a context for the interpretation of research findings.

Individual AWI-Gen centres are embarking on additional collaborative studies, which link into the AWI-Gen study. For example, the Agincourt centre embarked on harmonisation with the HAALSI study to enhance the objectives of both projects with a larger sample that extends the age group of the participants beyond 60 years and widens the data collection to include an extensive set of additional variables which address the process of aging, physical functioning, cognition, social variables and household data.

The partnership with the INDEPTH Network is an extraordinary opportunity to engage in the development of longitudinal studies and provides a rich context for future research far beyond our current objectives. INDEPTH has introduced a new Comprehensive Health and Epidemiological Surveillance System (CHESS) initiative [37] that requires the collection of biological data at field site laboratories and integrating these data with information collected via HDSS centres. CHESS will include detailed surveillance of risk factors, address the entire breadth of the rapidly transitioning burden of disease, including NCDs, and reference external causes and their associated morbidities. Individuals' biological and health diagnosis data will be linked with HDSS information. The requirements of CHESS will make it possible for many HDSS centres to have the requisite infrastructure to participate in an expanded AWI-Gen project.

Given the multidisciplinary partnerships both within and connected to AWI-Gen, and its footprint on the African continent, we are creating opportunities for CE and for interactions with health policy makers to address the health needs in the regions and to contribute to improved health management.

*How to contact us:* Potential collaborators should contact the AWI-Gen PI (Michèle Ramsay) or co-PI (Osman Sankoh).

## References

[1] UN. Sustainable Development Goals. 2015 (http://www.un.org/sustainabledevelopment/sustainable-development-goals/). Accessed 12 December 2015.

[2] WHO. Noncommunicable diseases country profiles 2014. 2014 (http://www.who.int/nmh/publications/ncd-profiles-2014/en/). Accessed 9 December 2015.

[3] Mensah GA. Descriptive epidemiology of cardiovascular risk factors and diabetes in sub-Saharan Africa. Progress in Cardiovascular Diseases 2013; 56: 240–250. 24267431 10.1016/j.pcad.2013.10.014 PMC11646150

[4] Dalal S, Non-communicable diseases in sub-Saharan Africa: what we know now. International Journal of Epidemiology 2011; 40: 885–901. 21527446 10.1093/ije/dyr050

[5] Crowther NJ, Norris SA. The current waist circumference cut point used for the diagnosis of metabolic syndrome in sub-Saharan African women is not appropriate. PLoS ONE 2012; 7: e48883. 23145009 10.1371/journal.pone.0048883 PMC3493601

[6] Ramsay M, Sankoh O, As members of the AWIGs, the HAC. African partnerships through the H3Africa Consortium bring a genomic dimension to longitudinal population studies on the continent. International Journal of Epidemiology 2015; 45: 305–308. 26659658 10.1093/ije/dyv187 PMC5841636

[7] Derra K, Profile: Nanoro Health and Demographic Surveillance system. International Journal of Epidemiology 2012; 41: 1293–1301. 23045201 10.1093/ije/dys159

[8] Oduro AR, Profile of the Navrongo health and demographic surveillance system. International Journal of Epidemiology 2012; 41: 968–976. 22933645 10.1093/ije/dys111

[9] Beguy D, Health & demographic surveillance system profile: the Nairobi urban health and demographic surveillance system (NUHDSS). International Journal of Epidemiology 2015; 44: 462–471. 25596586 10.1093/ije/dyu251

[10] Kahn K, Profile: Agincourt health and socio-demographic surveillance system. International Journal of Epidemiology 2012; 41: 988–1001. 22933647 10.1093/ije/dys115 PMC3429877

[11] Alberts M, Health & demographic surveillance system profile: the Dikgale health and demographic surveillance system. International Journal of Epidemiology 2015; 44: 1565–1571. 26275454 10.1093/ije/dyv157

[12] Richter L, Cohort profile: Mandela's children: the 1990 Birth to Twenty study in South Africa. International Journal of Epidemiology 2007; 36: 504–511. 17355979 10.1093/ije/dym016 PMC2702039

[13] Jaff NG, Body composition in the study of women entering and in endocrine transition (SWEET): a perspective of African women who have a high prevalence of obesity and HIV infection. Metabolism 2015; 64: 1031–1041. 26031506 10.1016/j.metabol.2015.05.009

[14] Beraldo RA, Proposed ratios and cutoffs for the assessment of lipodystrophy in HIV-seropositive individuals. European Journal of Clinical Nutrition 2015; 69: 274–278. 25074393 10.1038/ejcn.2014.149

[15] Kahn K, Validation and application of verbal autopsies in a rural area of South Africa. Tropical Medicine & International Health 2000; 5: 824–831. 11123832 10.1046/j.1365-3156.2000.00638.x

[16] Gauderman WJ, Morrison JM. QUANTO 1.1: a computer program for power and sample size calculations for genetic-epidemiology studies. 2006 (http://biostats.usc.edu/software). Accessed 19 September 2016.

[17] Gurdasani D, The African Genome Variation Project shapes medical genetics in Africa. Nature 2015; 517: 327–332. 25470054 10.1038/nature13997 PMC4297536

[18] Schlebusch CM, Soodyall H. Extensive population structure in San, Khoe, and mixed ancestry populations from southern Africa revealed by 44 short 5-SNP haplotypes. Human Biology 2012; 84: 695–724. 23959644 10.3378/027.084.0603

[19] Lachance J, Evolutionary history and adaptation from high-coverage whole-genome sequences of diverse African hunter-gatherers. Cell 2012; 150: 457–469. 22840920 10.1016/j.cell.2012.07.009 PMC3426505

[20] May A, Genetic diversity in black South Africans from Soweto. BMC Genomics 2013; 14: 644. 24059264 10.1186/1471-2164-14-644 PMC3850641

[21] Chimusa ER, A genomic portrait of haplotype diversity and signatures of selection in indigenous southern African populations. PLoS Genetics 2015; 11: e1005052. 25811879 10.1371/journal.pgen.1005052 PMC4374865

[22] Ramsay M. Africa: continent of genome contrasts with implications for biomedical research and health. FEBS Letters 2012; 586: 2813–2819. 22858376 10.1016/j.febslet.2012.07.061

[23] Ramsay M, Ethical issues in genomic research on the African continent: experiences and challenges to ethics review committees. Human Genomics 2014; 8: 15. 25145346 10.1186/s40246-014-0015-x PMC4420849

[24] de Vries J, The H3Africa policy framework: negotiating fairness in genomics. Trends in Genetics 2015; 31: 117–119. 25601285 10.1016/j.tig.2014.11.004 PMC4471134

[25] Tekola-Ayele F, Adeyemo AA, Rotimi CN. Genetic epidemiology of type 2 diabetes and cardiovascular diseases in Africa. Progress in Cardiovascular Diseases 2013; 56: 251–260. 24267432 10.1016/j.pcad.2013.09.013 PMC3840391

[26] The H3Africa Consortium, Research capacity. Enabling the genomic revolution in Africa. Science 2014; 344: 1346–1348. 24948725 10.1126/science.1251546 PMC4138491

[27] Harris PA, Research electronic data capture (REDCap) – a metadata-driven methodology and workflow process for providing translational research informatics support. Journal of Biomedical Informatics 2009; 42: 377–381. 18929686 10.1016/j.jbi.2008.08.010 PMC2700030

[28] The Ark. Centre for Genetic Origins of Health and Disease. 2014 (http://www.gohad.uwa.edu.au/enabling-resources/study-manager-and-lims-the-ark).

[29] Peprah E, Building a platform to enable NCD research to address population health in Africa: CVD working group discussion at the sixth H3Africa consortium meeting in Zambia. Global Heart 2016; 11: 165–170. 27102038 10.1016/j.gheart.2015.11.002 PMC4904225

[30] Hamdy O, Porramatikul S, Al-Ozairi E. Metabolic obesity: the paradox between visceral and subcutaneous fat. Current Diabetes Reviews 2006; 2: 367–373. 18220642 10.2174/1573399810602040367

[31] Schleinitz D, The genetics of fat distribution. Diabetologia 2014; 57: 1276–1286. 24632736 10.1007/s00125-014-3214-z

[32] Fox CS, Genome-wide association for abdominal subcutaneous and visceral adipose reveals a novel locus for visceral fat in women. PLoS Genetics 2012; 8: e1002695. 22589738 10.1371/journal.pgen.1002695 PMC3349734

[33] Gao C, A comprehensive analysis of common and rare variants to identify adiposity loci in hispanic Americans: the IRAS Family Study (IRASFS). PLoS ONE 2015; 10: e0134649. 26599207 10.1371/journal.pone.0134649 PMC4658008

[34] van der Merwe MT, Evidence for insulin resistance in black women from South Africa. International Journal of Obesity and Related Metabolic Disorders 2000; 24: 1340–1346. 11093297 10.1038/sj.ijo.0801416

[35] Goedecke JH, Differential effects of abdominal adipose tissue distribution on insulin sensitivity in black and white South African women. Obesity (Silver Spring) 2009; 17: 1506–1512. 19300428 10.1038/oby.2009.73

[36] Dillon DG, Association of HIV and ART with cardiometabolic traits in sub-Saharan Africa: a systematic review and meta-analysis. International Journal of Epidemiology 2013; 42: 1754–1771. 24415610 10.1093/ije/dyt198 PMC3887568

[37] Sankoh O, INDEPTH Network. CHESS: an innovative concept for a new generation of population surveillance. Lancet Global Health 2015; 3: e742. 26511039 10.1016/S2214-109X(15)00180-1

